# Crystal structure of bis­(thio­cyanato-κ*S*)bis­(thio­urea-κ*S*)mercury(II)

**DOI:** 10.1107/S2056989015000584

**Published:** 2015-01-17

**Authors:** A. Baskaran, K. Rajarajan, M. NizamMohideen, P. Sagayaraj

**Affiliations:** a Department of Physics, Rajeswari Vedachalam Government Arts College, Chengalpet 603 001, India; b Department of Physics, The New College (Autonomous), Chennai 600 014, India; c Department of Physics, Loyola College (Autonomous), Chennai 600 034, India

**Keywords:** crystal structure, thio­urea, thio­cyanate, mercury(II), mol­ecular complex, hydrogen bonding

## Abstract

In the title complex, [Hg(NCS)_2_(CH_4_N_2_S)_2_], the Hg^II^ atom is four-coordinated having an irregular four-coordinate geometry composed of four thione S atoms of two thio­cyanate groups and two thio­urea groups. The S—Hg—S angles are 172.02 (9)° for the *trans*-thio­cyanate S atoms and 90.14 (5)° for the *cis*-thio­urea S atoms. The mol­ecular structure is stabilized by an intra­molecular N—H⋯S hydrogen bond, which forms an *S*(6) ring motif. In the crystal, mol­ecules are linked by a number of N—H⋯N and N—H⋯S hydrogen bonds, forming a three-dimensional framework. The first report of the crystal structure of this compound appeared in 1966 [Korczynski (1966 ▸). *Rocz. Chem.* **40**, 547–569] with an extremely high *R* factor of 17.2%, and no mention of how the data were collected.

## Related literature   

For literature on thio­urea- and thio­cyanate-based metal–organic crystalline materials and their derivatives, see: Ramesh *et al.* (2012 ▸); Shihabuddeen Syed *et al.* (2013 ▸). For the concept of hard and soft acids and bases, see: Ozutsumi *et al.* (1989 ▸); Bell *et al.* (2001 ▸). For the crystal structures of similar compounds, see: Nawaz *et al.* (2010 ▸); Safari *et al.* (2009 ▸); Shihabuddeen Syed *et al.* (2013 ▸). For the first report of the crystal structure of the title compound, see: Korczynski (1966 ▸).
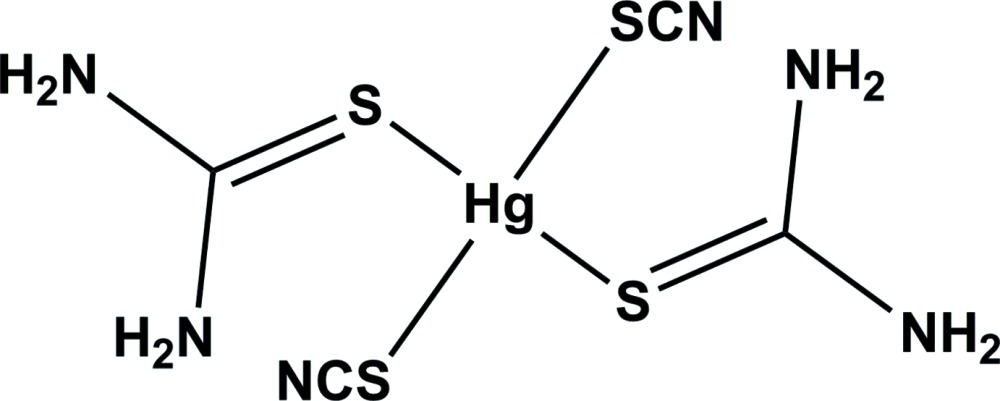


## Experimental   

### Crystal data  

[Hg(NCS)_2_(CH_4_N_2_S)_2_]
*M* *_r_* = 468.99
Orthorhombic, 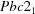

*a* = 8.5359 (5) Å
*b* = 9.0337 (5) Å
*c* = 15.7575 (10) Å
*V* = 1215.07 (12) Å^3^
*Z* = 4
Mo *K*α radiation
μ = 13.33 mm^−1^
*T* = 293 K
0.20 × 0.20 × 0.15 mm

### Data collection  

Bruker Kappa APEXII CCD diffractometer
Absorption correction: multi-scan (*SADABS*; Sheldrick, 2004 ▸) *T*_min_ = 0.176, *T*_max_ = 0.240
19798 measured reflections
2397 independent reflections
2158 reflections with *I* > 2σ(*I*)
*R*_int_ = 0.058

### Refinement  

*R*[*F* ^2^ > 2σ(*F* ^2^)] = 0.029
*wR*(*F* ^2^) = 0.075
*S* = 1.15
2397 reflections
137 parameters
1 restraint
H-atom parameters constrained
Δρ_max_ = 1.44 e Å^−3^
Δρ_min_ = −1.03 e Å^−3^
Absolute structure: Flack (1983 ▸), 1149 Freidel pairs.
Absolute structure parameter: 0.034 (12)

### 

Data collection: *APEX2* (Bruker, 2004 ▸); cell refinement: *APEX2* and *SAINT* (Bruker, 2004 ▸); data reduction: *SAINT* and *XPREP* (Bruker, 2004 ▸); program(s) used to solve structure: *SHELXS97* (Sheldrick, 2008 ▸); program(s) used to refine structure: *SHELXL97* (Sheldrick, 2015 ▸); molecular graphics: *ORTEP-3 for Windows* (Farrugia, 2012 ▸) and *PLATON* (Spek, 2009 ▸); software used to prepare material for publication: *WinGX* (Farrugia, 2012 ▸) and *PLATON*.

## Supplementary Material

Crystal structure: contains datablock(s) global, I. DOI: 10.1107/S2056989015000584/su5058sup1.cif

Structure factors: contains datablock(s) I. DOI: 10.1107/S2056989015000584/su5058Isup2.hkl

**Fig. 1.**
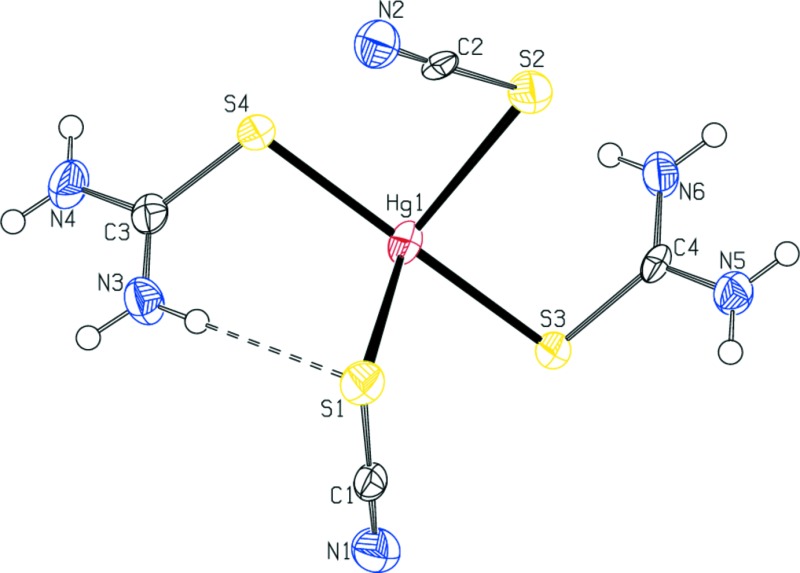
A view of the molecular structure of the title complex, with atom labelling. Displacement ellipsoids are drawn at the 50% probability level. The intramolecular N—H···S hydrogen bond is shown as a double dashed line (see Table 1 for details).

**Fig. 2.**
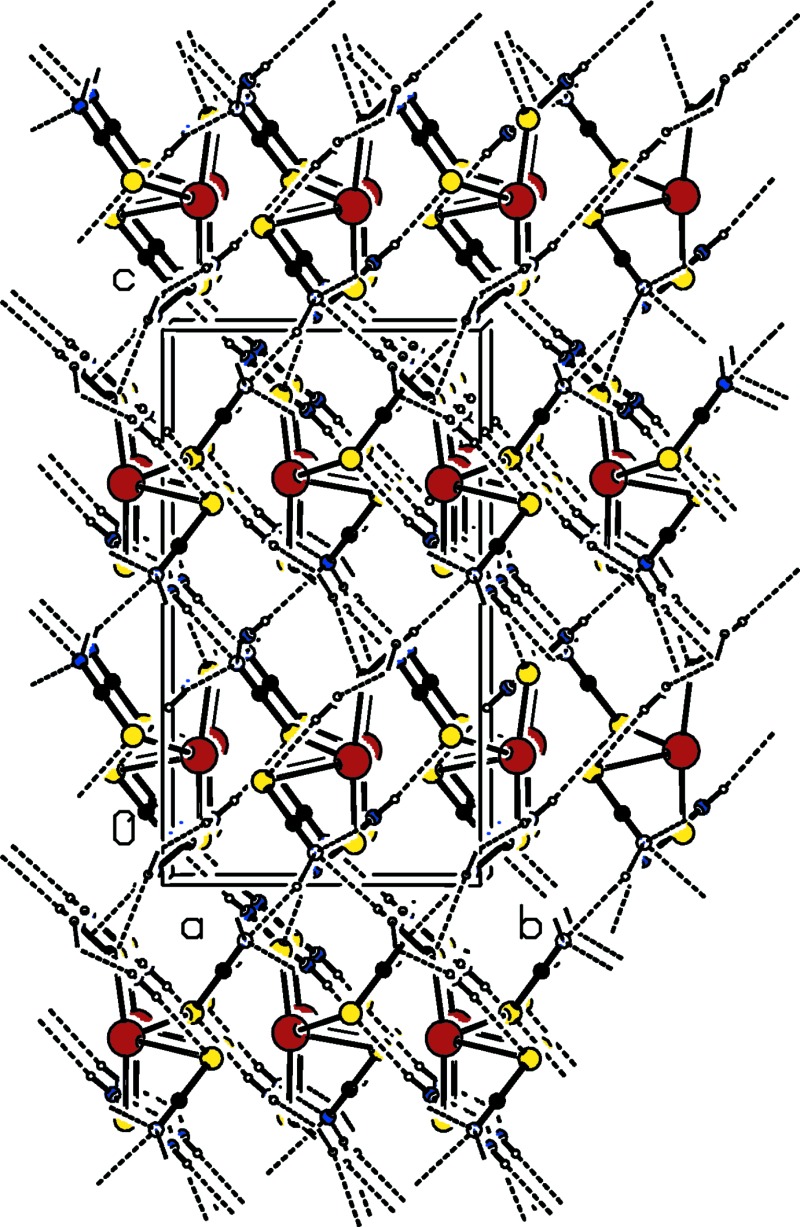
The crystal packing of the title complex, viewed along the a axis. Hydrogen bonds are shown as dashed lines (see Table 1 for details).

. DOI: 10.1107/S2056989015000584/su5058fig1.tif
A view of the mol­ecular structure of the title complex, with atom labelling. Displacement ellipsoids are drawn at the 50% probability level. The intra­molecular N—H⋯S hydrogen bond is shown as a double dashed line (see Table 1 for details).

a . DOI: 10.1107/S2056989015000584/su5058fig2.tif
The crystal packing of the title complex, viewed along the *a* axis. Hydrogen bonds are shown as dashed lines (see Table 1 for details).

CCDC reference: 1043131

Additional supporting information:  crystallographic information; 3D view; checkCIF report

## Figures and Tables

**Table 1:** Hydrogen-bond geometry (, )

*D*H*A*	*D*H	H*A*	*D* *A*	*D*H*A*
N3H3*B*S1	0.86	2.55	3.404(7)	174
N3H3*A*N2^i^	0.86	2.37	3.103(10)	143
N4H4*A*N2^i^	0.86	2.17	2.952(10)	151
N4H4*B*N1^ii^	0.86	2.56	3.085(11)	121
N5H5*A*N1^iii^	0.86	2.26	3.025(10)	149
N5H5*A*S4^iv^	0.86	2.80	3.384(7)	126
N5H5*B*N2^v^	0.86	2.21	3.025(10)	158
N6H6*A*N1^iii^	0.86	2.25	3.019(10)	149
N6H6*B*S2^vi^	0.86	2.56	3.419(8)	172

Symmetry codes: (i) 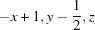; (ii) 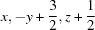; (iii) 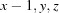; (iv) 
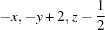
; (v) 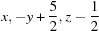; (vi) 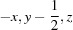.

## supplementary crystallographic information

### S1. Synthesis and crystallization

A mixture of thio­urea, ammonium thio­cyanate and mercury (II) chloride were dissolved in aqueous solution in the molar ratio 2:2:1 and thoroughly mixed for 1 h to obtain a homogeneous mixture. The solution was allowed to evaporate slowly at ambient temperature. Colourless block-like crystals were obtained in a week.

### S2. Refinement

All the H atoms were positioned geometrically with N—H = 0.86 Å and constrained to ride on their parent atoms with U_iso_(H) = 1.2U_eq_(N).

### S3. Comment

This work is part of a research project concerning the investigation of thiourea (N_2_H_4_CS) and thiocyanate (SCN) based metal organic crystalline materials and their derivatives (Ramesh *et al.*, 2012; Shihabuddeen Syed *et al.*, 2013). Transition metal thiourea and thiocyanate coordination complexes are candidate materials for device applications including their nonlinear optical properties. As ligands, both thiourea and thiocyanate are interesting due to their potential formation of metal coordination complexes as they exhibit multifunctional coordination modes due to the presence of 'S' and 'N' donor atoms. With reference to the hard and soft acids and bases) concept (Ozutsumi *et al.*, 1989; Bell *et al.*, 2001), the soft cations show a pronounced affinity for coordination with the softer ligands, while hard cations prefer coordination with harder ligands. Several crystallographic reports about mercury(II) complexes usually consist of discrete monomeric molecules with tetrahedral (somewhat distorted) coordination environments around mercury(II) (Nawaz *et al.*, 2010). Herein, we report on the synthesis and crystal structure of the title complex.

The title monomeric complex is composed of two thiocyanate and two thiourea ligands coordinated to the Hg atom via the softer thione S atom (Fig. 1). The four-coordinate mercury atom adopts a severely distorted tetrahedral geometry. The S—Hg—S angles are S3-Hg1-S4 = 172.02 (9) ° for the *trans* thiocyanate S atoms and S1-Hg1-S2 = 90.14 (5) ° for the *trans* thiourea S atoms. The bond distances Hg1-S3 and Hg1-S4 are 2.390 (3) and 2.381 (3) Å, respectively, while bond distances Hg1—S1 and Hg1—S2 are 3.064818) and 3.0836 (18) Å, respectively. Bond distances and angles are in agreement with those reported for related compounds (Shihabuddeen Syed *et al.*, 2013; Safari *et al.*, 2009; Nawaz *et al.*, 2010). The SCN moiety is planar [to within 0.007 (1) Å with the C-N and C-S bond lengths corresponding to the values intermediate between single and double bonds. The S2-C2-N2 and S1-C1-N1 units are nearly linear with bond angles of 178.5 (7) and 179.4 (8)°, respectively. The compound is closely related with (thiocyanato-*k*S)tris(thiourea-*k*S)mercury(II) chloride (Shihabuddeen Syed *et al.*, 2013). The molecular structure is stabilized by intramolecular an N-H···S hydrogen bond, which forms an S(6) ring motif (Fig. 1 and Table 1).

In the crystal, molecules are connected via N-H···N hydrogen bonds, involving the thiourea NH_2_ H atoms and the thiocyanate N atom (Fig. 2 and Table 1). This gives rise to the formation of a three-dimensional framework which is reinforced by N-H···S hydrogen bonds (Fig. 2 and Table 1).

The first report of the crystal structure of the title compound appeared in 1966 (Korczynski, 1966) with an extremely high R factor of 17.2 %, and no mention of how the data were collected.

### Crystal data

**Table:**

[Hg(NCS)_2_(CH_4_N_2_S)_2_]	*F*(000) = 872
*M**_r_* = 468.99	*D*_x_ = 2.564 Mg m^−^^3^
Orthorhombic, *P**b**c*2_1_	Mo *K*α radiation, λ = 0.71073 Å
Hall symbol: P 2c -2b	Cell parameters from 2397 reflections
*a* = 8.5359 (5) Å	θ = 2.4–31.2°
*b* = 9.0337 (5) Å	µ = 13.33 mm^−^^1^
*c* = 15.7575 (10) Å	*T* = 293 K
*V* = 1215.07 (12) Å^3^	Block, colourless
*Z* = 4	0.20 × 0.20 × 0.15 mm

### Data collection

**Table:**

Bruker Kappa APEXII CCD diffractometer	2397 independent reflections
Radiation source: fine-focus sealed tube	2158 reflections with *I* > 2σ(*I*)
Graphite monochromator	*R*_int_ = 0.058
ω and φ scans	θ_max_ = 26.0°, θ_min_ = 2.4°
Absorption correction: multi-scan (*SADABS*; Sheldrick, 2004)	*h* = −10→10
*T*_min_ = 0.176, *T*_max_ = 0.240	*k* = −11→11
19798 measured reflections	*l* = −19→19

### Refinement

**Table:**

Refinement on *F*^2^	Hydrogen site location: inferred from neighbouring sites
Least-squares matrix: full	H-atom parameters constrained
*R*[*F*^2^ > 2σ(*F*^2^)] = 0.029	*w* = 1/[σ^2^(*F*_o_^2^) + (0.0237*P*)^2^ + 4.6956*P*] where *P* = (*F*_o_^2^ + 2*F*_c_^2^)/3
*wR*(*F*^2^) = 0.075	(Δ/σ)_max_ = 0.001
*S* = 1.15	Δρ_max_ = 1.44 e Å^−^^3^
2397 reflections	Δρ_min_ = −1.02 e Å^−^^3^
137 parameters	Extinction correction: *SHELXL97* (Sheldrick, 2008), Fc^*^=kFc[1+0.001xFc^2^λ^3^/sin(2θ)]^-1/4^
1 restraint	Extinction coefficient: 0.0082 (3)
Primary atom site location: structure-invariant direct methods	Absolute structure: Flack (1983), 1149 Freidel pairs.
Secondary atom site location: difference Fourier map	Absolute structure parameter: 0.034 (12)

### Special details

**Table:**

Geometry. Bond distances, angles etc. have been calculated using the rounded fractional coordinates. All su's are estimated from the variances of the (full) variance-covariance matrix. The cell esds are taken into account in the estimation of distances, angles and torsion angles
Refinement. Refinement of *F*^2^ against ALL reflections. The weighted *R*-factor *wR* and goodness of fit *S* are based on *F*^2^, conventional *R*-factors *R* are based on *F*, with *F* set to zero for negative *F*^2^. The threshold expression of *F*^2^ > σ(*F*^2^) is used only for calculating *R*-factors(gt) *etc*. and is not relevant to the choice of reflections for refinement. *R*-factors based on *F*^2^ are statistically about twice as large as those based on *F*, and *R*- factors based on ALL data will be even larger.

### Fractional atomic coordinates and isotropic or equivalent isotropic displacement parameters (Å^2^)

**Table:**

	*x*	*y*	*z*	*U*_iso_*/*U*_eq_	
Hg1	0.25569 (4)	0.89378 (3)	0.72851 (8)	0.0374 (1)	
S1	0.4564 (2)	1.1691 (2)	0.69569 (13)	0.0336 (6)	
S2	−0.0283 (2)	1.0924 (2)	0.76929 (13)	0.0334 (6)	
S3	0.2017 (3)	0.8900 (3)	0.57967 (14)	0.0329 (7)	
S4	0.3068 (3)	0.8610 (3)	0.87589 (15)	0.0353 (7)	
N1	0.6053 (9)	1.0000 (9)	0.5700 (5)	0.041 (3)	
N2	0.0984 (9)	1.2628 (8)	0.9017 (5)	0.037 (3)	
N3	0.6051 (8)	0.9346 (7)	0.8444 (5)	0.034 (2)	
N4	0.5630 (8)	0.7508 (9)	0.9391 (4)	0.039 (2)	
N5	−0.0507 (8)	1.0248 (7)	0.5262 (4)	0.031 (2)	
N6	−0.0982 (7)	0.8404 (8)	0.6204 (5)	0.032 (2)	
C1	0.5444 (9)	1.0700 (9)	0.6213 (5)	0.026 (2)	
C2	0.0478 (9)	1.1927 (9)	0.8463 (5)	0.025 (2)	
C3	0.5073 (8)	0.8485 (9)	0.8853 (5)	0.026 (2)	
C4	0.0009 (9)	0.9200 (8)	0.5772 (4)	0.022 (2)	
H3A	0.70430	0.92670	0.85300	0.0410*	
H3B	0.57020	0.99910	0.80900	0.0410*	
H4A	0.66240	0.74400	0.94710	0.0460*	
H4B	0.50010	0.69370	0.96640	0.0460*	
H5A	−0.14960	1.04050	0.52150	0.0370*	
H5B	0.01460	1.07740	0.49760	0.0370*	
H6A	−0.19710	0.85630	0.61570	0.0380*	
H6B	−0.06470	0.77190	0.65370	0.0380*	

### Atomic displacement parameters (Å^2^)

**Table:**

	*U*^11^	*U*^22^	*U*^33^	*U*^12^	*U*^13^	*U*^23^
Hg1	0.0319 (2)	0.0600 (2)	0.0205 (2)	−0.0029 (1)	−0.0073 (2)	0.0032 (3)
S1	0.0359 (11)	0.0344 (10)	0.0304 (10)	−0.0005 (9)	−0.0003 (8)	−0.0009 (9)
S2	0.0319 (11)	0.0370 (10)	0.0314 (11)	0.0017 (9)	0.0016 (9)	−0.0026 (9)
S3	0.0231 (10)	0.0548 (14)	0.0209 (11)	0.0038 (9)	−0.0006 (9)	−0.0002 (9)
S4	0.0243 (11)	0.0616 (14)	0.0200 (11)	0.0005 (10)	0.0013 (9)	−0.0005 (10)
N1	0.035 (4)	0.051 (5)	0.038 (4)	0.000 (4)	0.003 (4)	−0.005 (4)
N2	0.039 (4)	0.035 (4)	0.037 (5)	0.002 (3)	−0.002 (3)	0.002 (3)
N3	0.026 (4)	0.033 (4)	0.044 (4)	−0.001 (3)	−0.005 (3)	0.011 (3)
N4	0.041 (4)	0.044 (4)	0.031 (4)	0.010 (3)	−0.005 (3)	0.009 (3)
N5	0.031 (4)	0.031 (4)	0.030 (4)	0.000 (3)	−0.003 (3)	0.006 (3)
N6	0.026 (3)	0.036 (4)	0.033 (4)	−0.001 (3)	−0.007 (3)	0.007 (3)
C1	0.024 (4)	0.032 (4)	0.022 (4)	−0.004 (3)	−0.004 (3)	0.008 (3)
C2	0.025 (4)	0.036 (4)	0.015 (4)	0.003 (3)	0.005 (3)	0.011 (3)
C3	0.028 (4)	0.031 (4)	0.019 (4)	0.008 (3)	−0.002 (3)	−0.010 (3)
C4	0.028 (4)	0.026 (4)	0.012 (4)	0.001 (3)	−0.005 (3)	−0.001 (3)

### Geometric parameters (Å, º)

**Table:**

Hg1—S1	3.0641 (18)	N4—C3	1.313 (11)
Hg1—S2	3.0836 (18)	N5—C4	1.318 (9)
Hg1—S3	2.390 (3)	N6—C4	1.302 (10)
Hg1—S4	2.381 (3)	N3—H3A	0.8600
S1—C1	1.655 (8)	N3—H3B	0.8600
S2—C2	1.648 (8)	N4—H4A	0.8600
S3—C4	1.736 (8)	N4—H4B	0.8600
S4—C3	1.722 (7)	N5—H5A	0.8600
N1—C1	1.151 (11)	N5—H5B	0.8600
N2—C2	1.162 (11)	N6—H6A	0.8600
N3—C3	1.310 (10)	N6—H6B	0.8600
			
S1—Hg1—S2	90.14 (5)	C3—N3—H3A	120.00
S1—Hg1—S3	87.35 (8)	C3—N3—H3B	120.00
S1—Hg1—S4	99.39 (8)	H3A—N3—H3B	120.00
S2—Hg1—S3	93.51 (8)	S3—C4—N5	117.1 (6)
S2—Hg1—S4	90.75 (8)	S3—C4—N6	122.9 (6)
S3—Hg1—S4	172.02 (9)	N5—C4—N6	119.9 (7)
Hg1—S1—C1	86.2 (3)	C3—N4—H4A	120.00
Hg1—S2—C2	99.4 (3)	C3—N4—H4B	120.00
Hg1—S3—C4	102.1 (2)	H4A—N4—H4B	120.00
Hg1—S4—C3	105.9 (3)	C4—N5—H5A	120.00
S1—C1—N1	179.4 (8)	C4—N5—H5B	120.00
S2—C2—N2	178.4 (8)	H5A—N5—H5B	120.00
S4—C3—N3	123.5 (6)	C4—N6—H6A	120.00
S4—C3—N4	117.4 (6)	C4—N6—H6B	120.00
N3—C3—N4	119.1 (7)	H6A—N6—H6B	120.00
			
S2—Hg1—S1—C1	−145.2 (3)	S2—Hg1—S3—C4	−26.2 (3)
S3—Hg1—S1—C1	−51.7 (3)	S1—Hg1—S4—C3	−55.8 (3)
S4—Hg1—S1—C1	124.0 (3)	S2—Hg1—S4—C3	−146.1 (3)
S1—Hg1—S2—C2	−56.6 (3)	Hg1—S3—C4—N6	−53.0 (7)
S3—Hg1—S2—C2	−144.0 (3)	Hg1—S3—C4—N5	129.8 (5)
S4—Hg1—S2—C2	42.8 (3)	Hg1—S4—C3—N4	−139.9 (6)
S1—Hg1—S3—C4	−116.2 (3)	Hg1—S4—C3—N3	42.8 (8)

### Hydrogen-bond geometry (Å, º)

**Table:**

*D*—H···*A*	*D*—H	H···*A*	*D*···*A*	*D*—H···*A*
N3—H3*B*···S1	0.86	2.55	3.404 (7)	174
N3—H3*A*···N2^i^	0.86	2.37	3.103 (10)	143
N4—H4*A*···N2^i^	0.86	2.17	2.952 (10)	151
N4—H4*B*···N1^ii^	0.86	2.56	3.085 (11)	121
N5—H5*A*···N1^iii^	0.86	2.26	3.025 (10)	149
N5—H5*A*···S4^iv^	0.86	2.80	3.384 (7)	126
N5—H5*B*···N2^v^	0.86	2.21	3.025 (10)	158
N6—H6*A*···N1^iii^	0.86	2.25	3.019 (10)	149
N6—H6*B*···S2^vi^	0.86	2.56	3.419 (8)	172

Symmetry codes: (i) −*x*+1, *y*−1/2, *z*; (ii) *x*, −*y*+3/2, *z*+1/2; (iii) *x*−1, *y*, *z*; (iv) −*x*, −*y*+2, *z*−1/2; (v) *x*, −*y*+5/2, *z*−1/2; (vi) −*x*, *y*−1/2, *z*.
