# Supplementary figures and images for: Crystal structure of bis­(thio­cyanato-κS)bis­(thio­urea-κS)mercury(II)
Source: Acta Crystallogr E Crystallogr Commun. 2015 Jan 17;71(Pt 2):m28–9. doi: 10.1107/S2056989015000584 (PMC4384628; doi:10.1107/S2056989015000584)

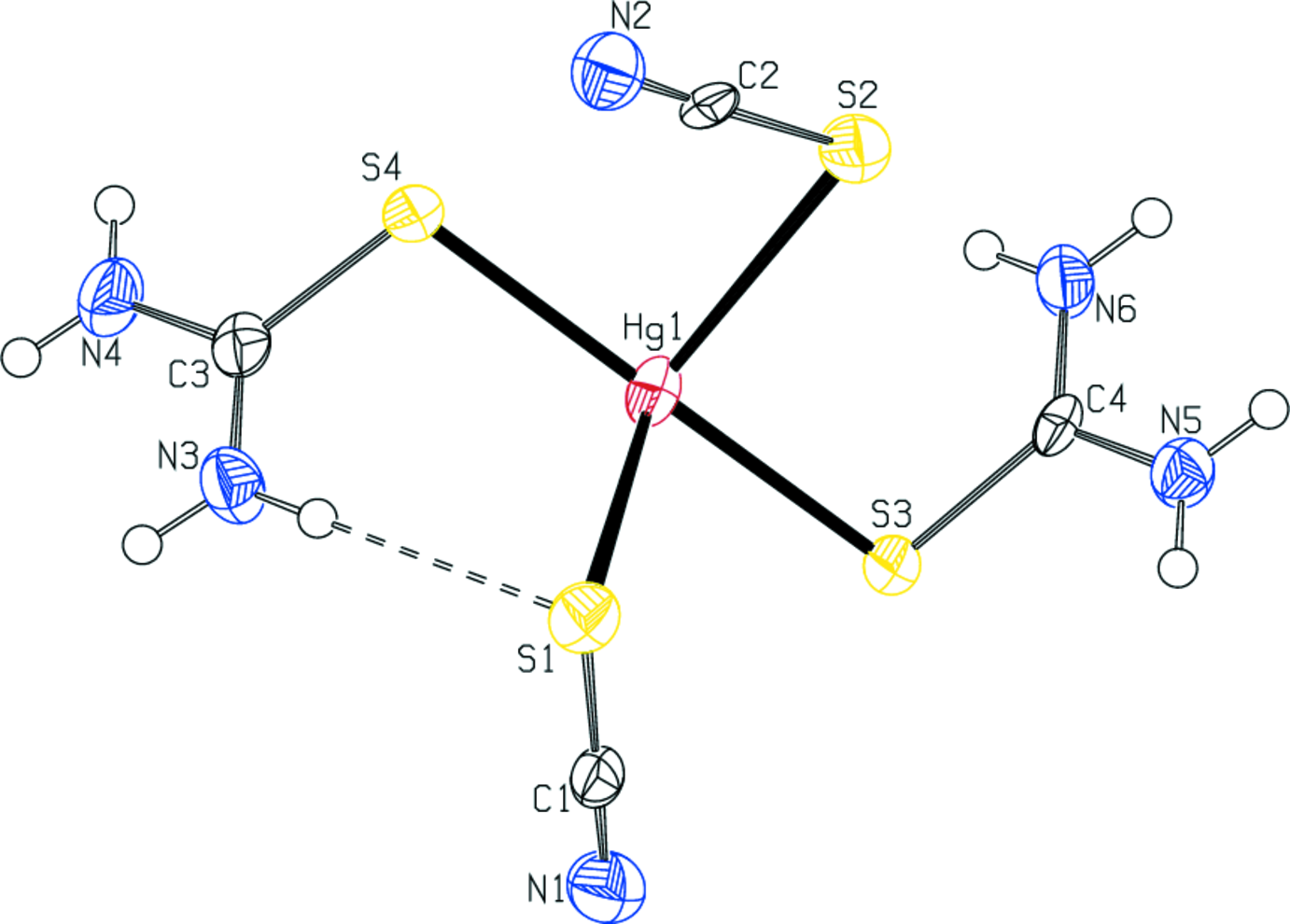

Supplement: Supplementary file 3 [file e-71-00m28-fig1.tif]

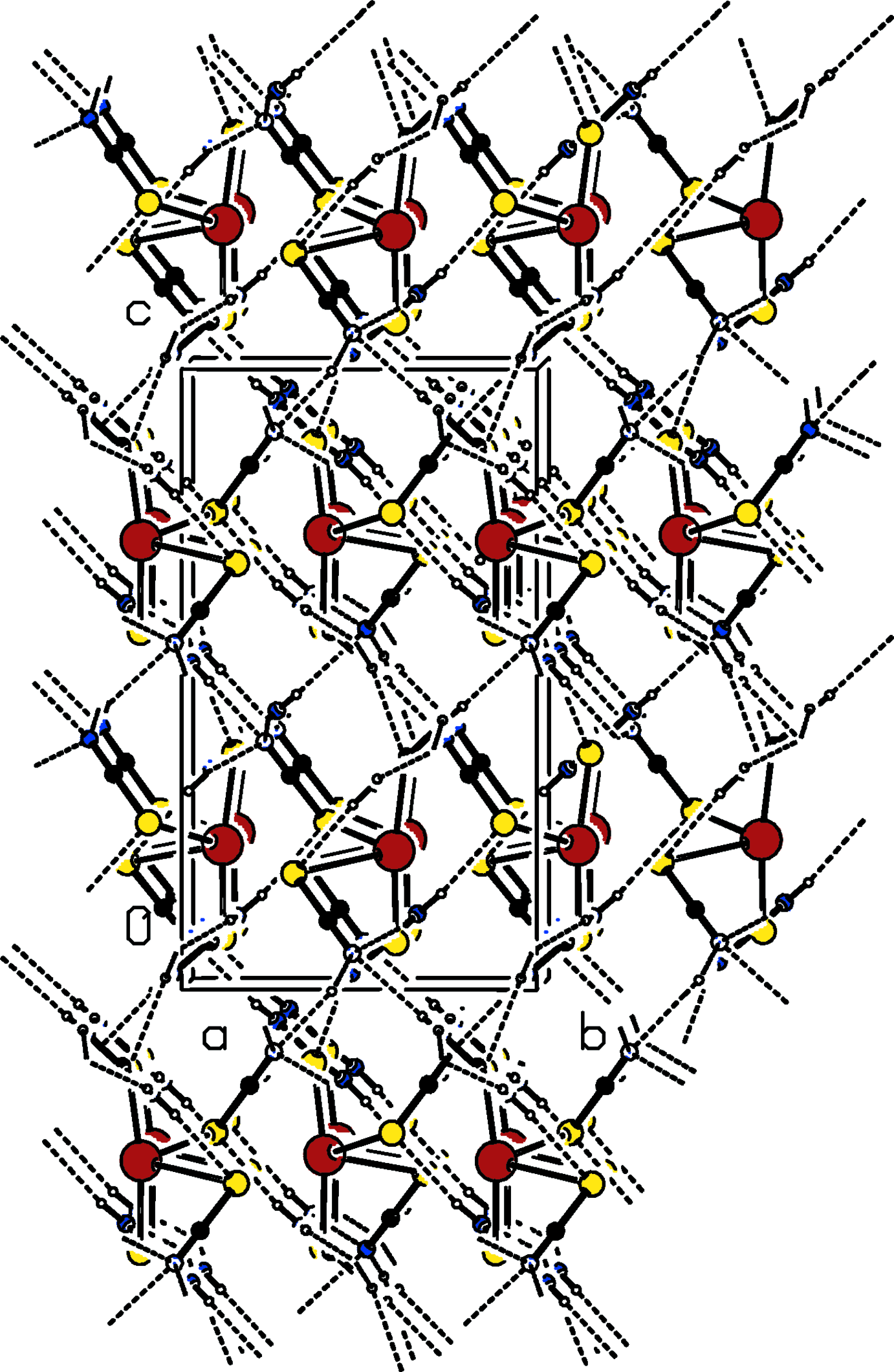

Supplement: Supplementary file 4 [file e-71-00m28-fig2.tif]
